# Adiponectin and Leptin Induce VCAM-1 Expression in Human and Murine Chondrocytes

**DOI:** 10.1371/journal.pone.0052533

**Published:** 2012-12-19

**Authors:** Javier Conde, Morena Scotece, Verónica López, Rodolfo Gómez, Francisca Lago, Jesús Pino, Juan Jesús Gómez-Reino, Oreste Gualillo

**Affiliations:** 1 NEIRID Lab (NeuroEndocrine Interaction in Rheumatology and Inflammatory Diseases), SERGAS, Santiago University Clinical Hospital, Institute of Medical Research (IDIS), Santiago de Compostela, Spain; 2 Division of Rheumatology, Fundación Jiménez Diaz, Madrid, Spain; 3 Research Laboratory 7 (Molecular and Cellular Cardiology), SERGAS, Santiago University Clinical Hospital, Institute of Medical Research (IDIS), Santiago de Compostela, Spain; 4 Division of Orthopaedics Surgery and Traumatology, SERGAS, Santiago University Clinical Hospital, Santiago de Compostela, Spain; University of Minho, Portugal

## Abstract

**Background:**

Osteoarthritis (OA) and rheumatoid arthritis (RA), the most common rheumatic diseases, are characterized by irreversible degeneration of the joint tissues. There are several factors involved in the pathogenesis of these diseases including pro-inflammatory cytokines, adipokines and adhesion molecules.

**Objective:**

Up to now, the relationship between adipokines and adhesion molecules at cartilage level was not explored. Thus, the aim of this article was to study the effect of leptin and adiponectin on the expression of VCAM-1 in human and murine chondrocytes. For completeness, intracellular signal transduction pathway was also explored.

**Methods:**

VCAM-1 expression was assessed by quantitative RT-PCR and western blot analysis upon treatment with leptin, adiponectin and other pertinent reagents in cultured human primary chondrocytes. Signal transduction pathways have been explored by using specific pharmacological inhibitors in the adipokine-stimulated human primary chondrocytes and ATDC5 murine chondrocyte cell line.

**Results:**

Herein, we demonstrate, for the first time, that leptin and adiponectin increase VCAM-1 expression in human and murine chondrocytes. In addition, both adipokines have additive effect with IL-1β. Finally, we demonstrate that several kinases, including JAK2, PI3K and AMPK are at a play in the intracellular signalling of VCAM-1 induction.

**Conclusions:**

Taken together, our results suggest that leptin and adiponectin could perpetuate cartilage-degrading processes by inducing also factors responsible of leukocyte and monocyte infiltration at inflamed joints.

## Introduction

Osteoarthritis (OA), one of the most common rheumatic diseases, is a pathology characterized by irreversible joint cartilage destruction. Biochemical, genetic and mechanical factors [1] affect the onset and progression of OA. The role of obesity in OA is known from time. Actually, the dysfunction of adipose tissue associated with altered adipokine secretion pattern is emerging as relevant factor that affect joint structures [2], [3], [4].

Chondrocytes are the unique cell type of adult human articular cartilage capable to maintain extracellular matrix components integrity and turnover [5]. In osteoarthritis, due to abnormal environmental insults, chondrocytes produce a wide range of inflammatory mediators leading cartilage loss [6] including adipokines and vascular cell adhesion molecules (VCAM) [7], [8].

VCAM-1 is an inducible surface glycoprotein that belongs to immunoglobulin gene superfamily (IgSF) [9]. VCAM-1 serves as surface ligand for VLA-4 (α_4_β_1_) integrin [10] and this adhesion molecule plays a main role in the adhesion of lymphocytes to endothelium in the site of inflammation [11].

VCAM-1 expression is increased in RA and OA synovial tissue [12], [13]. Synovial fibroblast and chondrocytes express VCAM-1 [7], [14] and pro-inflammatory cytokines such as IL-1β and TNF-α are able to up-regulate VCAM-1 expression in primary cultures of human articular chondrocytes [7]. VCAM-1 might contribute to adhesion of T-lymphocytes to chondrocytes, and thus participate in host defense mechanisms during inflammatory joint conditions such as rheumatoid arthritis or osteoarthritis and/or after cartilage transplantation [7], [15]. Recently, it has been described that VCAM-1 is a strong and independent predictor of the risk of knee and hip joint replacement due to severe OA [16]. In addition, serum level of soluble VCAM-1 was associated with hand OA [17].

In earlier studies we demonstrated that adipokines are novel and potent factors able to modulate chondrocytes physiology. Thus, the aim of this study was to describe the effect of different adipokines (adiponectin leptin and visfatin) on the expression of VCAM-1 in chondrocytes and to elucidate the potential intracellular mechanism involved in the signalling pathway triggered by adipokines.

## Materials and Methods

### Reagents

All culture reagents were from Sigma (MO, USA), and Lonza, (Switzerland). For RT-PCR, a First Strand Kit, Master mix, primers for VCAM-1 and GAPDH were purchased from SABiosciences (MD, USA). Nucleospin kits for RNA and protein isolation were from Macherey-Nagel (Germany). Mouse and human recombinant leptin, mouse and human recombinant IL-1β, tyrphostin AG490, LY294002, PD098059 and compound C were from Sigma (MO, USA), and recombinant mouse and human adiponectin and visfatin from BioVendor (Germany).

### Cell Culture and Treatments

Human primary chondrocytes and the murine ATDC-5 cell line culture were developed as previously described [18], [19], [20]. Briefly, normal human articular cartilage samples were obtained from the knee joints of 10 patients undergoing knee amputations for peripheral vascular disease or total knee replacement surgery (with permission from the local ethics committee). Cartilage samples were obtained from the joint area of minimal load with normal morphologic examination (i.e., no change in color and no fibrillation). Human chondrocytes were cultured in DMEM/Ham’s F12 medium supplemented with 10% of fetal bovine serum, L-glutamine, and antibiotics (50 units/ml penicillin and 50 µg/ml streptomycin). Cells were seeded in monolayer up to the high density and used freshly in order to avoid dedifferentiation.

Murine chondrogenic cell line ATDC-5, passage 30–50 (purchased from RIKEN Cell Bank, Tsukuba, Japan), were cultured in DMEM–Ham’s F-12 medium supplemented with 5% fetal bovine serum, 10 µg/ml human transferrin, 3×10^−8^ M sodium selenite, and antibiotics (50 units/ml penicillin and 50 µg/ml streptomycin). ATDC-5 cells were differentiated into mature chondrocytes and hypertrophic chondrocytes. Briefly, cells were seeded at a density of 6×10^3^/cm^2^ in 6-well plates with the ATDC-5 standard media supplemented with insulin (10 µg/mL). The differentiation media was replaced every two days for 14 days. On day 15, the culture medium was switched to α-MEM up to day 21 in order to obtain hypertrophic cells. Differentiation was qualitatively characterized by increased formation of cell nodules and enhanced staining with Alcian blue, which are indicative of proteoglycan accumulation. In other experiments (data not shown), differentiation was further analyzed by sequential increase in the levels of type II collagen, aggrecan and type X collagen mRNA, as previously published [18], [19].

For RT-PCR and western blot, cells were seeded in P6 multiwell plates until complete adhesion and then incubated overnight in serum-free conditions. Cells were treated with mouse or human IL-1β (10 ng/ml), mouse or human leptin (400 or 800 nM), mouse or human adiponectin (0.1, 1, 5 and 10 µg/ml), mouse or human visfatin (500 ng/ml). Specific pharmacological inhibitors were added 1 h before stimulation: tyrphostin AG490 (10 µM) for JAK2 inhibition, PD098059 (30 µM) for mitogen-activated protein kinase kinase (MEK1) inhibition, LY294002 (10 µM) for phosphatidylinositol 3-kinase (PI3K) inhibition and compound C (10 µM) for AMPK inhibition.

### RNA Isolation and Real-time Reverse Transcription–polymerase Chain Reaction (RT-PCR)

Human and murine VCAM-1 mRNA levels were determined using SYBR Green–based quantitative PCR. RNA was extracted using a NucleoSpin kit, according to the manufacturer’s instructions. For cDNA synthesis, we performed a RT reaction with a SABiosciences First Strand Kit, using 1 µg of RNA. Next, real-time PCR was performed using specific primers (for human VCAM-1, 141 bp, PPH00623E, reference position 2879, GenBank accession no. NM_001078.2; for mouse VCAM-1, 146 bp, PPM03208B, reference position 2870, GenBank accession no. NM_011693.3; for mouse GAPDH, 140 bp, PPM02946E, reference position 309–328, GenBank accession no. NM_008084.2; for human GAPDH, 175 bp, PPH00150E, reference position 1287–1310, GenBank accession no. NM_002046.3) and Master mix (SABiosciences, MD, USA). All reagents used for RT-PCR were added at the concentrations provided by the manufacturer: 12.5 µL of Master mix, 10.5 µL of water and 1 µL of primers were used by each sample. Results of comparative real-time PCRs were analyzed using MxPro software (Stratagene, CA, USA).

### Western Blot Analysis

Proteins were extracted using a NucleoSpin kit, according to the manufacturer’s instructions; electrophoresis and blotting procedures have been described previously (4). Immunoblots were incubated with the appropriate antibody (anti-VCAM-1 diluted 1∶500, Santa Cruz, CA,USA; anti-phospho-JAK2 diluted 1∶1000, Cell Signalling, MA, USA; anti-JAK2 diluted 1∶1000, Cell Signalling, MA, USA; anti-phospho-PI3K diluted 1∶1000, Cell Signalling, MA, USA; anti-PI3K diluted 1∶1000, Cell Signalling, MA, USA; anti-phospho-AMPK diluted 1∶1000, Cell Signalling, MA, USA; anti-AMPK diluted 1∶1000, Cell Signalling, MA, USA) and visualized using an Immobilon Western kit (Millipore, MA, USA) and anti-goat (Santa Cruz, CA, USA) or anti-rabbit (GE Healthcare, UK) horseradish-peroxidise-labelled secondary antibody diluted 1∶2000. To confirm equal loading for each sample, after stripping in glycine buffer at pH3, membranes were reblotted with anti-actin antibody diluted 1∶5000 (Sigma, MO, USA). Autoradiographs were analyzed with an EC3 imaging system (UVP, CA, USA).

### Statistical Analysis

Data are reported as the mean ± SEM of at least 3 independent experiments. The comparison method for RT-PCR was performed as previously (18). Statistical analyses were performed by analysis of variance, followed by post-hoc comparison testing (using the unpaired *t*-test and Student-Newman-Keuls test) using the GraphPad Prism 4 computerized package (GraphPad Software). P values less than 0.05 were considered significant.

## Results

### VCAM-1 Induction by Cytokines and Adipokines in Human Primary Chondrocytes and ATDC-5 Chondrocytes

**Figure 1:**
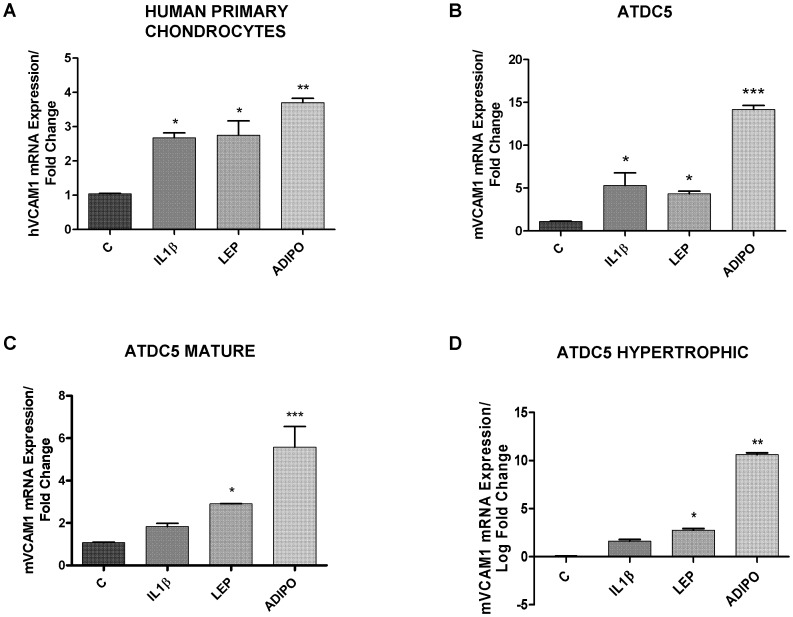
Determination of hVCAM-1 and mVCAM-1 mRNA expression by quantitative real-time PCR. **A**. hVCAM-1 expression after IL-1β (10 ng/ml), leptin (800 nM) and adiponectin (10 µg/ml) treatment in human primary chondrocytes, during 24 hours. **B,C,D**. mVCAM-1 expression after IL-1β (10 ng/ml), leptin (800 nM) and adiponectin (10 µg/ml) treatment in undifferentiated ATDC-5 cells (B), mature ATDC-5 cells (C) and hypertrophic ATDC-5 cells, during 24 hours.

As shown in figure 1, IL-1β (10 ng/ml), leptin (800 nM) and adiponectin (10 µM) are able to significantly induce the VCAM-1 mRNA expression in human primary chondrocytes (A) and in ATDC-5 chondrocytes at different stages of differentiation (B–C–D) after 24 hours treatment. To note, adiponectin was the most potent adipokine in inducing VCAM-1.

### VCAM-1 Induction by Adiponectin in ATDC5 Cell Line

**Figure 2:**
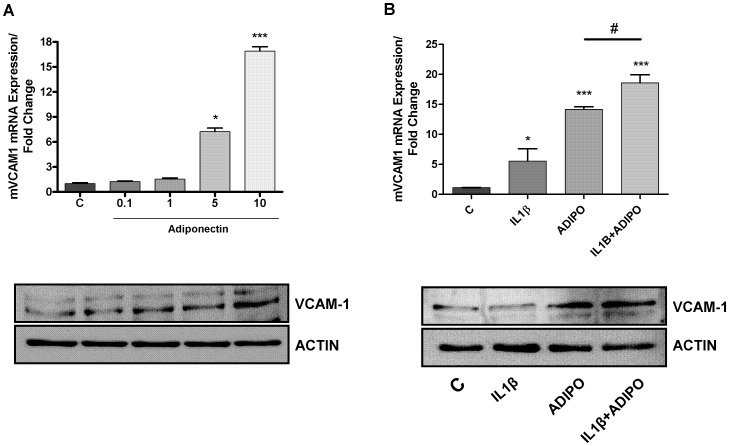
Determination of mVCAM-1 mRNA and protein expression by quantitative real-time PCR and western blot. **A**. mVCAM-1 expression after adiponectin (0.1, 1, 5, 10 µg/ml) treatment in ATDC-5 cell line, during 24 hours. **B**. mVCAM-1 expression after IL-1β (10 ng/ml), adiponectin (10 µg/ml) and the combination of IL-1β (10 ng/ml) plus adiponectin (10 µg/ml) challenge in ATDC-5 cell line, during 24 hours.

As shown in figure 2A, adiponectin was able to induce VCAM-1 expression in a dose- dependent manner.

When cells have been stimulated with a combination of IL-1β and adiponectin, the expression of VCAM-1 was significantly higher than in the cells stimulated with IL-1β or adiponectin alone (figure 2B).

These results were confirmed also in terms of protein expression (figure 2A–B low panels).

### Effect of the Specific Signalling Pathway Inhibitors on Adiponectin-induced VCAM-1 Expression

To gain further insights into the intracellular mechanism(s) responsible for VCAM-1 induction by adiponectin, we evaluated the effect of specific pharmacological inhibitors of relevant kinases such as MEK1, PI3K and AMPK.

**Figure 3:**
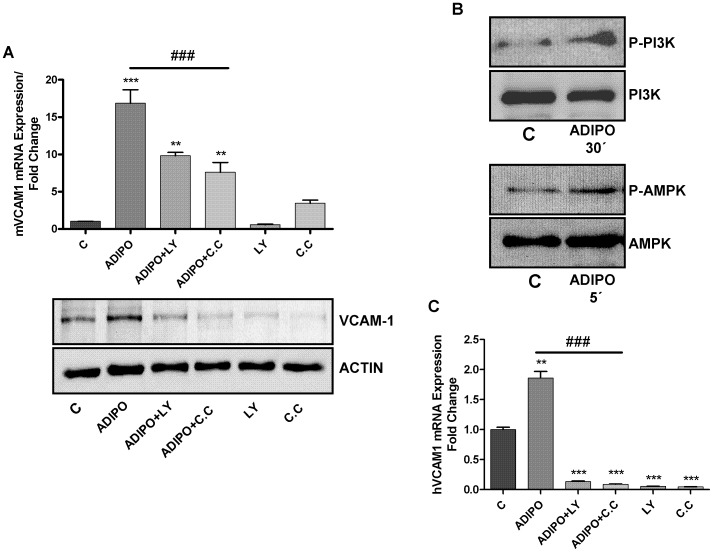
Determination of VCAM-1 mRNA and protein expression by quantitative real-time PCR and western blot. **A**. mVCAM-1 expression after 1 hour pre-treatment with LY294002 (10 µM) and compound C (10 µM), followed by a 24 hours of adiponectin (10 µg/ml) challenge in ATDC-5 cells. **B**. Determination of the phosphorylation of PI3K and AMPK by western blot. **C**. hVCAM-1 expression after 1 hour pre-treatment with LY294002 (10 µM) and compound C (10 µM), followed by a 24 hours of adiponectin (10 µg/ml) challenge in human primary chondrocytes.

As shown in figure 3A, addition of LY294002 and compound C (inhibitors of PI3K and AMPK respectively), one hour before adiponectin treatment, resulted in a significant decrease in mVCAM-1 expression in the ATDC-5 cell line (figure 3A).

The phosphorylation of PI3K and AMPK by adiponectin was confirmed by also western blot (figure 3B).

Inhibition of PI3K and AMPK by LY294002 and compound C respectively, also decreased adiponectin-induced hVCAM-1 in human primary chondrocytes (figure 3C).

### VCAM-1 Induction by Leptin in ATDC5 Cell Line

**Figure 4:**
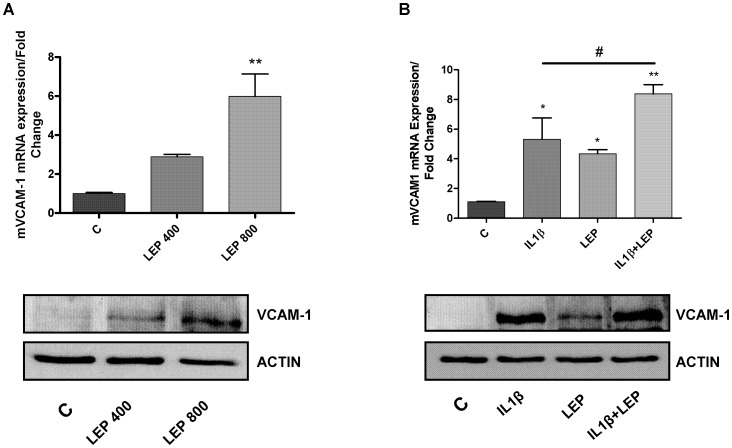
Determination of mVCAM-1 mRNA and protein expression by quantitative real-time PCR and western blot. **A.** mVCAM-1 expression after leptin (400 and 800 nM) treatment in ATDC-5 cell line, during 24 hours. B. mVCAM-1 mRNA expression after IL-1β (10 ng/ml), leptin (800 nM) and the combination of IL-1β (10 ng/ml) plus leptin (800 nM) challenge in ATDC-5 cell line, during 24 hours.

As shown in figure 4A, leptin was able to induce VCAM-1 expression in a dose- dependent manner.

When cells have been stimulated with a combination of IL-1β and leptin, the expression of VCAM-1 was significantly higher than the cells stimulated with IL-1β or leptin alone (figure 4B).

These results were also confirmed in terms of protein expression (figure 4A–B low panels).

### Effect of the Specific Signalling Pathway Inhibitors on Leptin-induced VCAM-1 Expression

To gain further insights into the intracellular mechanism(s) responsible for VCAM-1 induction by leptin, we evaluated the effect of specific pharmacological inhibitors of relevant kinases such as JAK2 and PI3K.

**Figure 5:**
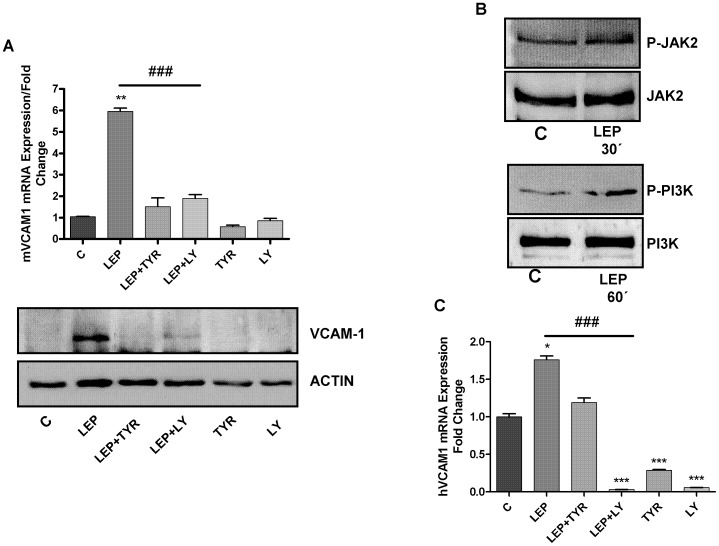
Determination of VCAM-1 mRNA and protein expression by quantitative real-time PCR and western blot. **A.** mVCAM-1 expression after 1 hour pre-treatment with tyrphostin AG490 (10 µM) and LY294002 (10 µM), followed by a 24 hours of leptin (800 nM) challenge in ATDC-5 cells. **B.** Determination of the phosphorylation of JAK2 and PI3K by western blot. **C**. hVCAM-1 expression after 1 hour pre-treatment with tyrphostin AG490 (10 µM) and LY294002 (10 µM), followed by a 24 hours of leptin (10 µg/ml) challenge in human primary chondrocytes.

As shown in figure 5A, cell stimulation with leptin in presence of tyrphostin AG490 and LY294002 (inhibitors of JAK2 and PI3K respectively), one hour before adipokine challenge, significantly decrease mVCAM-1 expression in the ATDC-5 cell line. The phosphorylation of JAK2 and PI3K by leptin was confirmed by western blot (figure 5B).

Inhibition of JAK2 and PI3K by tyrphostin AG490 and LY294002 respectively, also decreased leptin-induced hVCAM-1 in human primary chondrocytes (figure 5C).

## Discussion

Recently chondrocytes have been recognized to synthesize also adipokines such as adiponectin and leptin [21], [22]. This novel superfamily of metabolic factors mediates prevalently inflammatory processes at joint cartilage level. For instance, it has been reported that adiponectin induces NOS2, MMP-3, MMP-9 and IL-6 in chondrocytes [4]. Moreover adiponectin plasma levels are associated with markers of cartilage degradation and these levels are higher in patients with most severe OA [22] and adiponectin levels are increased in RA patients as well [23]. Similarly, leptin and IL-1 induce synergistically nitric oxide in chondrocytes [24]. The inflammatory environment that exists in the joint, produced in part by adipokines, generates changes in the synovium, including synovial hypertrophy and inflammatory cells infiltration [25].

Therefore extravasation of leukocytes from circulating blood to inflamed tissue is crucial in inflammatory processes and this complex event is regulated by adhesion molecules such as VCAM-1 [26]. The expression of this adhesion molecule affects the binding and recruitment of leucocytes into inflamed joints. For instance, antibody blockade of VCAM-1 decreased significantly the binding of lymphocytes to joint vessels [27]. Similarly, other author demonstrated that incubating synovial fluid with anti-VCAM-1 resulted in a significant inhibition of monocyte chemotaxis [28].

Due to the relevance of adipokines and VCAM-1 respectively in the joint inflammatory processes, we investigated, for the first time, the relationship between these factors. An earlier article reported the induction of VCAM-1 by IL-1β in chondrocytes [7]; thus, we have used the stimulation with this cytokine as our positive control of VCAM-1 induction. Our current study shows, for the first time, a clear VCAM-1 mRNA induction by leptin and adiponectin in human primary chondrocytes. Similar results were obtained in ATDC5 cell line, being adiponectin the most potent inductor, even more than IL-1β. The intracellular pathway/s involved in the adiponectin-induced VCAM-1 expression was, up to now, unknown. Thus we have explored, by using specific pharmacological inhibitors the potential involvement of several intracellular kinases. Our results clearly show that AMPK and PI3K are at play in the VCAM-1 induction by adiponectin. In the same way, JAK2 and PI3K are involved in VCAM-1 induction by leptin.

Moreover, in this study we observed that treatment with adiponectin or leptin in combination with IL-1β, resulted in an additive induction of VCAM-1. Noteworthy, our group demonstrated that leptin is able to synergize with IL-1β in the production of nitric oxide [24]. We also tested the effect of visfatin on VCAM-1 expression, and any modulation was observed neither in human primary chondrocytes nor in ATDC-5 cells (data not shown).

Several lines of evidence suggest that adipokines are clearly involved in degenerative joint disease such as RA and OA. This study extends our current knowledge of adipokine functions by specifically demonstrating that both leptin and adiponectin are able to induce VCAM-1 directly in cultured human and murine chondrocytes, thus contributing to a better understanding of disease etiology. So, it is reasonable to depict a scenario in which adipokines may perpetuate cartilage degrading processes by inducing also factors responsible of leukocyte and monocyte infiltration at inflamed joints.
